# Middle Miocene biogeographic connectivity between the Eastern Ghats and Nepal revealed by a new species of the Cyrtodactylus (Geckoella) nebulosus complex (Reptilia, Squamata) from Nepal

**DOI:** 10.3897/zookeys.1275.178507

**Published:** 2026-03-25

**Authors:** Bivek Gautam, Santosh Bhattarai, Bishal Prasad Neupane, Chiranjibi Prasad Pokheral, Tejas Thackeray, Akshay Khandekar, Vivek Phillip Cyriac, Ishan Agarwal

**Affiliations:** 1 Biodiversity Research and Conservation Society, Kathmandu, Nepal Department of Zoology, Shivaji University Kolhapur India; 2 Future Regions Research Centre, Federation University Australia, Gippsland Campus, Churchill 3842, Victoria, Australia Natural History Museum London United Kingdom; 3 Nepal Conservation and Research Center, Ratnanagar-06, Sauraha, Chitwan, Nepal Biodiversity Research and Conservation Society Kathmandu Nepal; 4 National Trust for Nature Conservation, Khumaltar, P.O.Box - 3712, Kathmandu, Nepal Nepal Conservation and Research Center Chitwan Nepal; 5 Thackeray Wildlife Foundation, Mumbai, 400051, India Future Regions Research Centre, Federation University Australia Victoria Australia; 6 Department of Zoology, Shivaji University, Kolhapur, 416004, India National Trust for Nature Conservation Kathmandu Nepal; 7 Natural History Museum, Cromwell Rd, South Kensington, London, SW7 5BD, UK Thackeray Wildlife Foundation Mumbai India

**Keywords:** Endemic species, Indian subcontinent, integrative taxonomy, mitochondrial DNA, taxonomy

## Abstract

A new species of ground-dwelling gecko of the genus Cyrtodactylus (Geckoella) is described from the low elevation Terai-Duar region of southeastern Nepal using molecular and morphological data. *Cyrtodactylus teraiensis***sp. nov**. is the first new species of the *C. nebulosus* species complex, the remaining members of which are distributed in the northern Eastern Ghats and Satpuras; a lectoype for *C. nebulosus* is also designated. The new species is the first *Geckoella* described from outside peninsular India and distributed north of the Indo-Gangetic Plains. *Cyrtodactylus teraiensis***sp. nov**. forms the deeply divergent sister taxon to Indian members of the *C. nebulosus* complex with 15.7–18.1% uncorrected mitochondrial sequence divergence from them, and 21.0–28.5% from other *Geckoella*. The new species is also recognised in tree-based delimitation methods and can be morphologically distinguished from other *Cyrtodactylus* and *Geckoella* species by a small body size (snout to vent length, SVL up to at least 45.5 mm), length of original tail < SVL, 16–18 rows of dorsal tubercles, 30–32 ventral scales across belly at midbody; dorsal colour pattern of four or five paired spots between neck and hindlimb insertions alternating with two or three much smaller paired spots. The divergence between *Cyrtodactylus teraiensis***sp. nov**. and Indian members of the *C. nebulosus* complex is estimated to have occurred in the Middle Miocene, and it may be that tropical forest expansion during the Mid-Miocene Climatic Optimum allowed dispersal across the Indo-Gangetic Plains.

## Introduction

The Indian subcontinent is a fascinating, diverse landscape — its unique geological history and current location at the juncture of biogeographic realms make it of particular interest to biogeographers (Mani 1974; Chatterjee et al. 2013). The Indian subcontinent is variously referred to as including India and Sri Lanka as well as parts of Bangladesh, Bhutan, Nepal, and Pakistan; we use the term to denote the boundary of the Indian Plate — that is all (mainland) areas from the Himalaya southward. This vast landscape spans large gradients in latitude, longitude, climate, and topography, and consists of peninsular India, the Himalaya including the eastern and western syntaxes in Pakistan and Indo-Burma, northeast India (and Bangladesh), the Indus Division (sensu Corbet and Hill 1992; Agarwal et al. 2014), the Indo-Gangetic Plains, as well as Sri Lanka, the Andaman and Nicobar Islands, and Lakshadweep.

The Indian subcontinent has high levels of diversity and endemism across taxonomic groups, within which peninsular India stands out for its particularly large number of endemic radiations (Karanth 2015). Peninsular India is dominated by the dry zone, with forests restricted to the Western Ghats, and to a lesser extent, the Eastern Ghats and other highland areas. The Indian Plate was ancestrally aseasonal and covered by tropical forest, and though the first indications of seasonality date back to the Eocene, the dry zone is relatively recent in origin and has expanded greatly since the late Miocene (Harris 2006; Ganjoo and Shaker 2007; Nelson 2007; Clift et al. 2008; Guo et al. 2008; Morley 2011, 2018; Molnar and Rajagopalan 2012; Licht et al. 2014). A result of this history is that many of the endemic radiations in peninsular India are of taxa restricted to cool habitats. Some of the gekkonid genera typical of cool, forested/ rocky and or upland habitats in peninsular India include *Calodactylodes* Strand, 1928; *Cnemaspis* Strauch, 1887; Cyrtodactylus (Geckoella) Gray, 1827; *Dravidogecko* Smith, 1993; the *prashadi* group of Indian *Hemidactylus* Goldfuss, 1820; and *Hemiphyllodactylus* Bleeker, 1860 (Smith 1935; Agarwal et al. 2019a, 2019b; Pal et al. 2021).

The mega-diverse genus *Cyrtodactylus* includes more than 380 species distributed from the Western Himalayas through South and Southeast Asia to the Western Pacific (Wood et al. 2012; Grismer et al. 2021; Uetz et al. 2022). There are a number of geographically circumscribed clades, among the most interesting of which is the ground-dwelling radiation of the subgenus *Geckoella* Gray, 1867 (Agarwal and Karanth 2015) in peninsular India and Sri Lanka (Smith 1935; Agarwal 2016; Agarwal et al. 2016, 2023a, 2023b; Narayanan et al. 2022). *Geckoella* corresponds to the *triedrus* clade within the *triedrus* group (Grismer et al. 2021) and includes 17 species including four species complexes. The *C. triedrus* (Günther, 1864) complex is restricted to Sri Lanka and includes three species, the *C. albofasciatus* (Boulenger, 1885) - *C. deccanensis* (Günther, 1864) complex has two species, the *C. collegalensis* (Beddome, 1870) complex contains ten species, and the *C. nebulosus* (Beddome, 1870) complex a single named species, in addition to the morphologically unique *C. jeyporensis* (Beddome, 1878) from the Eastern Ghats. Members of the *C. albofasciatus*- *C. deccanensis* complex are distributed in the Northern and Central Western Ghats; the *C. collegalensis* complex mainly occurs in southern India with one species distributed in central and western India and one in Sri Lanka; and the *C. nebulosus* complex is distributed in the Eastern Ghats and central India (Smith 1935; Somaweera and Somaweera 2009; Agarwal and Karanth 2015; Agarwal 2016; Agarwal et al. 2016, 2023a, 2023b; Narayanan et al. 2022).

*Cyrtodactylus nebulosus* was described by Beddome (1870) from the “Golcondah hills near Vizagapatam [= Visakhapatnam, Andhra Pradesh]… at 2,000–3,500 feet [~600–1100 m] elevation”. Like other members of the subgenus *Geckoella*, *C. nebulosus* is ground-dwelling, nocturnal, and largely restricted to hilly and or forest habitats. It has been recorded from the Eastern Ghats in northern Andhra Pradesh and Odisha as far north as Kharagpur in southern West Bengal, as well as the Satpuras in Chhattisgarh and Madhya Pradesh (Agarwal 2007; Sur et al. 2007; Agarwal and Karanth 2015). Molecular phylogenetic data demonstrated that *C. nebulosus* is a species complex (Agarwal and Karanth 2015), although no additional species have been described thus far.

**Figure 1.**
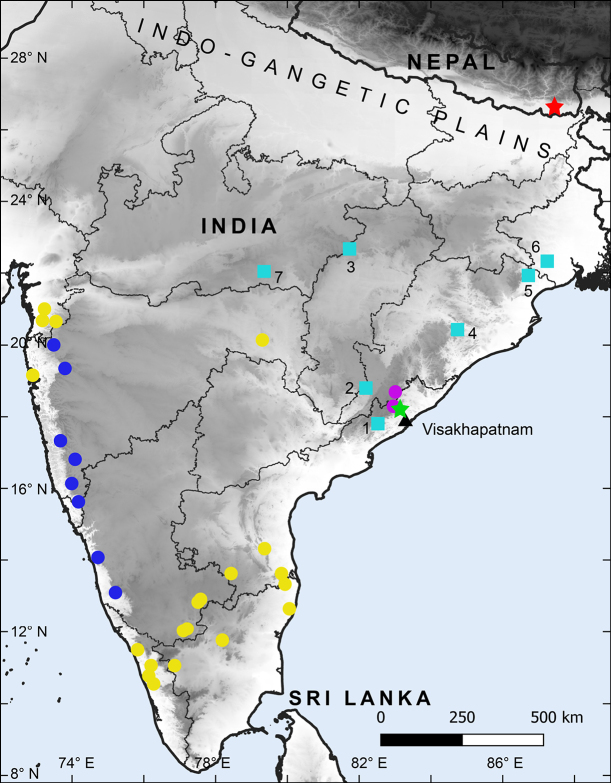
Map showing sampled localities of Indian Cyrtodactylus (Geckoella): blue circles *C. albofasciatus*-*C. deccanensis* complex; yellow circles *C. collegalensis* complex; pink circles *C. jeyporensis*; green star approximate type locality of *C. nebulosus* and red star type locality of *C. teraiensis* sp. nov.; blue squares *C. nebulosus* complex (1, Narsipatnam; 2, Baripada; 3, Tikarpada; 4, Gupteswar; 5, Amarkantak; 6, Kharagpur; 7, Pench; 6, 7 literature records). Visakhapatnam is marked by a black triangle.

We present the first record of a member of the subgenus *Geckoella* from outside peninsular India (Fig. 1). We collected two specimens of a ground-dwelling *Cyrtodactylus* with rounded dorsal tubercles from low elevation, subtropical, Sal-dominated (*Shorea robusta* Roth) forest in the Terai-Duar of Nepal. These specimens morphologically resembled members of the *Cyrtodactylus nebulosus* complex – and phylogenetic data place them as the sister taxon to the entire complex from India. We describe this Nepalese population as *Cyrtodactylus teraiensis* sp. nov. We also use this opportunity to designate a lectotype for *C. nebulosus* and provide a diagnosis for the species based on the type series and topotypical material.

## Materials and methods

### Field survey and sample collection

*Cyrtodactylus* specimens were surveyed during opportunistic surveys in lowland Terai-Duar forests as part of a larger project on squamates of Nepal. Two specimens of the new species were encountered that were hand-collected, photographed while alive, and later euthanised. Liver tissue samples were collected from both euthanised specimens and stored in 100% ethanol until they could be transported for subsequent long-term storage at -20 °C. Whole specimens were fixed in 8–14% formalin for ~12–24 hours and later transferred to 70% ethanol after being thoroughly washed. Collection permits for this work were issued by the Nepal Government Department of National Parks and Wildlife Conservation and Department of Forests and Soil Conservation (see acknowledgements). Specimens are deposited in the Natural History Museum, Kathmandu, Nepal (**NHM**) originally bearing Bivek Gautam field numbers (**BG**).

### Molecular data and phylogenetic analyses

We extracted DNA from thawed liver tissues of both Nepalese *Cyrtodactylus* samples using the Qiagen DNeasy kit and targeted the mitochondrial ND2 gene (1038 nucleotides, nt) with the primers MetF1 (L4437) and H5540 (Macey et al. 1997) for amplification and sequencing. Extractions were conducted at the National Trust for Nature Conservation (**NTNC**)-Biodiversity Conservation Center, Sauraha, Chitwan District, Bagmati Province, Nepal; PCR and Sanger sequencing were outsourced to Barcode Biosciences (Bangalore). Chromatograms were assembled using Chromas 2.6.6 (Technelisium, Australia; http://technelysium.com.au/wp/chromas/) and new sequences were combined with published sequences for all members of the *triedrus* clade (Grismer et al. 2021), with *Cyrtodactylus fraenatus* (Günther) + *C. soba* Batuwita & Bahir used as the outgroup (Table 1).

**Table 1.** List of sequences used in this study with museum number, locality, and GenBank accession number. Museum and voucher abbreviations: ADS = Anselm de Silva field series; AK = Akshay Khandekar field series; BG = Bivek Gautam field series; BNHS = Bombay Natural History Society, Mumbai; CES/ESV = Centre for Ecological Sciences, Bangalore; DMSSK = D.M.S. Suranjan Karunarathna field series; IAG = Ishan Agarwal field series; JB = John Boone private collection; NCBS and NRC (National Centre for Biological Sciences, Bangalore); ZSI = Zoological Survey of India, Kolkata.

Species	Museum No.	Locality	GenBank Accession Numbers	
*Cyrtodactylus teraiensis* sp. nov.	NHM 2025/390 (BG-81266)	Nepal: Koshi Province, Morang District, Bhaunne	PX879553	*C. nebulosus* complex
*Cyrtodactylus teraiensis* sp. nov.	NHM 2025/391 (BG-81277)	Nepal: Koshi Province, Morang District, Bhaunne	PX879553	
*Cyrtodactylus nebulosus*	NRC-AA-9627	India: Andhra Pradesh, Visakhapatnam District, Araku	PX896570	
	(CES09/1201)			
*Cyrtodactylus nebulosus*	NRC-AA-9629	India: Andhra Pradesh, Visakhapatnam District, near Araku	PX896571	
	(CES09/1203)			
*Cyrtodactylus nebulosus*	NRC-AA-9616	India: Andhra Pradesh, Visakhapatnam District, Araku-S.Peta Road	PX896572	
	(CES09/1508)			
*Cyrtodactylus cf. nebulosus* 1	CES09/1351	India: Andhra Pradesh, Visakhapatnam District, Narsipatnam	KM878618	
*Cyrtodactylus cf. nebulosus* 2	CES09/1118	India: Orissa, Mayurbhanj District, Baripada	KM878620	
*Cyrtodactylus cf. nebulosus* 3	CES09/1119	India: Orissa, Mayurbhanj District, Tikarpada	KM878619	
*Cyrtodactylus cf. nebulosus* 4	CES09/1205	India: Orissa, Koraput District, Gupteswar	KM878621	
*Cyrtodactylus cf. nebulosus* 5	CES09/1374	India: Madhya Pradesh, Anuppur District, Amarkantak	KM878622	
*Cyrtodactylus jeyporensis*	CES09/1206	India: Orissa, Koraput District, Deomali	KM878617	*C. jeyporensis*
*Cyrtodactylus jeyporensis*	CES09/1356	India: Andhra Pradesh, Visakhapatnam District, Araku Valley	KM878616	
*Cyrtodactylus aravindi*	ZSI-R 28275	India: Tamil Nadu, Kanyakumari District, Nagercoil	OP131039	*C. collegalensis* complex
*Cyrtodactylus aravindi*	ZSI-R 28280	India: Tamil Nadu, Kanyakumari District, Thuckalay	OP131040	
*Cyrtodactylus chengodumalaensis*	NRC-AA-1163 (CES09/1410)	India: Kerala, Thrissur District, Mannuthy	OP271668	
*Cyrtodactylus chengodumalaensis*	NRC-AA-1166 (CES09/1411)	India: Kerala, Thrissur District, Mannuthy	OP271669	
*Cyrtodactylus chengodumalaensis*	BNHS 2812 (CES09/1412)	India: Kerala, Calicut District, Narayamkulam	OP271670	
*Cyrtodactylus chengodumalaensis*	NRC-AA-1161 (CES09/1449)	India: Kerala, Malappuram District, Kumaragiri Estate	OP271671	
*Cyrtodactylus chengodumalaensis*	BNHS 2817 (AK 665)	India: Kerala, Palakkad District, Cheeni Paara	OP271672	
*Cyrtodactylus collegalensis*	CES09/1403	India: Tamil Nadu, Salem District, Mettur Taluk	KX632365	
*Cyrtodactylus collegalensis*	CES09/1442	India: Karnataka, Chamarajanagar District, MM Hills	KX632362	
*Cyrtodactylus collegalensis*	CES09/1443	India: Karnataka, Chamarajanagar District, Kollegal Taluk	KX632363	
*Cyrtodactylus collegalensis*	CES09/1444	India: Karnataka, Chamarajanagar District, MM Hills	KM878627	
*Cyrtodactylus collegalensis*	CES09/1463	India: Karnataka, Chamarajanagar District, MM Hills	KX632364	
*Cyrtodactylus irulaorum*	NRC-AA-1266	India; Tamil Nadu, Chengalpattu District, near Chengalpet	OQ674252	
*Cyrtodactylus irulaorum*	NRC-AA-1270	India; Tamil Nadu, Chengalpattu District, near Chengalpet	OQ674253	
*Cyrtodactylus irulaorum*	NRC-AA-1271	India; Tamil Nadu, Tiruvallur District, near Thervoykandigai	OQ674254	
*Cyrtodactylus relictus*	NRC-AA-1275	India; Andhra Pradesh, Chittoor District, Kambakkam Durg	OQ674255	
*Cyrtodactylus relictus*	NRC-AA-1276	India; Andhra Pradesh, Nellore District, near Bhairavakona Waterfalls	OQ674256	
*Cyrtodactylus relictus*	NRC-AA-1274	India; Andhra Pradesh, Nellore District, near Bhairavakona Waterfalls	OQ674257	
*Cyrtodactylus rishivalleyensis*	ESV 104	India: Andhra Pradesh, Chittoor District	KX698080	
	(CES09/1245)			
*Cyrtodactylus rishivalleyensis*	ESV 103	India: Andhra Pradesh, Chittoor District	KX698081	
	(CES09/1452)			
*Cyrtodactylus speciosus*	CES09/1405	India: Tamil Nadu, Coimbatore District, Coimbatore North Taluk	KM878623	
*Cyrtodactylus speciosus*	CES09/1249	India: Tamil Nadu, Salem District, below Yercaud	KM878629	
*Cyrtodactylus srilekhae*	ESV 101	India: Karnataka, Bengaluru Rural District, near Thathaguni	KX698082	
	(CES09/1432)			
*Cyrtodactylus srilekhae*	ESV 102	India: Karnataka, Bengaluru Rural District, Nandi Hills	KX698083	
	(CES09/1461)			
*Cyrtodactylus srilekhae*	CES09/1536	India: Karnataka, Tumkur District, Devarayandurga	KX698084	
*Cyrtodactylus varadgirii*	BNHS 1848	India: Maharashtra, Mumbai District, Mumbai	KX632366	
*Cyrtodactylus varadgirii*	BNHS 1849	India: Maharashtra, Mumbai District, Mumbai	KX632367	
*Cyrtodactylus varadgirii*	BNHS 2099	India: Gujarat, Navsari District, Vansda	KX632368	
*Cyrtodactylus varadgirii*	CES09/1381	India: Gujarat, Navsari District, Chikhli	KM878612	
*Cyrtodactylus varadgirii*	CES09/1433	India: Gujarat, Navsari District, Kangvai	KX632369	
*Cyrtodactylus yakhuna*	DMSSK 159	Sri Lanka: Polonnaruwa District, Giritale Forest	MW713942	
*Cyrtodactylus albofasciatus*	CES09/1109	India: Karnataka, Chikmagalur District, Kudremukh	KM878626	*C. albofasciatus*-deccanensis complex
*Cyrtodactylus albofasciatus*	CES09/1117	India: Karnataka, Shimoga District, Nagavalli	KM878625	
*Cyrtodactylus cf. albofasciatus*	CES09/1391	India: Maharashtra, Kolhapur District, Patgaon	KM878610	
*Cyrtodactylus cf. albofasciatus*	CES09/1418	India: Goa, North Goa District, Chorla Ghat	KM878611	
*Cyrtodactylus cf. albofasciatus*	JB7	India: captive (from Indian stock)	JX440521	
*Cyrtodactylus deccanensis*	CES09/1112	India: Maharashtra, Satara District, Bhairavgad	KM878615	
*Cyrtodactylus deccanensis*	CES09/1243	India: Maharashtra, Thane District, Malshej Ghat	KM878614	
*Cyrtodactylus deccanensis*	CES09/1380	India: Maharashtra, Nashik District, Amboli	KM878613	
*Cyrtodactylus cf. deccanensis*	CES09/1396	India: Maharashtra, Kolhapur District, Panhala	KM878628	
*Cyrtodactylus punctatus*	DMSSK 180	Sri Lanka: Matale District, Knuckles	MW713941	*C. triedrus* complex
*Cyrtodactylus triedrus*	DMSSK 011	Sri Lanka: Kandy District, Dunumadalawa Forest	MW713937	
*Cyrtodactylus vedda*	ADS35	Sri Lanka: Yakkunehela	JX440522	
*Cyrtodactylus fraenatus*	DMSSK 046	Sri Lanka: Kandy District, Gannoruwa	MW713940	Outgroups
*Cyrtodactylus soba*	DMSSK 124	Sri Lanka: Matale District, Knuckles	MW713938	

Sequence alignment was carried out using CLUSTAL W (Thompson et al. 1994) in MEGA 5.2 (Tamura et al. 2011), with translation to amino acids to verify protein-coding sequences. Uncorrected p-distance was calculated in MEGA 5.2 using the pairwise deletion option (Table 2). The best partitioning scheme and models of sequence evolution were selected in PartitionFinder 2.1.1 (Lanfear et al. 2012) for Maximum Likelihood (ML) analyses in RaXML HPC 8.2.12 (Stamatakis 2014) and Bayesian Inference (BI) in MrBayes 3.2.7 (Ronquist and Huelsenbeck 2003). The best partitioning scheme was by codon position (cp), with GTR+G for cp1 and cp2 and GTR+I for cp3. A ML phylogeny was reconstructed using the ND2 data partitioned by codon position in RAxML as implemented in the raxmlGUI 2.0.10 (Edler et al. 2020), applying the GTR + G model, 1000 thorough bootstraps and 10 independent starting trees. Partitioned BI were conducted in MrBayes using the best-fit partition scheme with two parallel runs and four chains each (one cold and three hot) run for 2,000,000 generations sampling every 200 generations. Convergence was determined based on the standard deviation of split frequencies (<<0.01) and the first 25% of trees were discarded as burn-in. A Maximum Clade Credibility tree was generated using TreeAnnotator 1.10.4 (Drummond et al. 2012).

**Table 2.** Uncorrected pairwise genetic distance (mitochondrial ND2 gene, 1038 nt) between species of the *Cyrtodactylus nebulosus* complex and other *Geckoella*. Along diagonal, maximum variation within species.

	Species	1	2	3	4	5	6	7	8	9	10
1	*C. teraiensis* sp. nov.	0.1									
2	*C. nebulosus*	17.3	0.8								
3	*C. cf. nebulosus* 1	17.6	6.0	-							
4	*C. cf. nebulosus* 2	18.1	12.3	13.0	-						
5	*C. cf. nebulosus* 3	15.7	9.9	8.8	9.0	-					
6	*C. cf. nebulosus* 4	15.7	11.8	11.0	12.1	8.6	-				
7	*C. cf. nebulosus* 5	16.4	12.9	9.9	12.3	9.0	4.8	-			
8	*C. jeyporensis*	21.0	21.4	20.7	24.0	22.3	21.2	22.5	-		
9	*C. collegalensis* complex	23.1	22.8	22.6	24.6	23.8	24.0	24.8	23.7	-	
10	*C. albofasciatus*-*C. deccanensis* complex	28.5	29.1	27.8	28.2	28.6	28.0	28.4	31.6	29.0	-
11	*C. triedrus* complex	21.4	23.4	22.2	23.2	23.3	23.3	23.8	25.7	26.3	28.3

### Species delimitation

We conducted species delimitation with one distance-based and two tree-based methods. As just a single species is recognised within the *nebulosus* complex, we used the lowest pairwise distance between members of its sister clade, the *C. collegalensis* complex that can be distinguished based on morphology (= 8.7% uncorrected ND2 sequence divergence; Agarwal et al. 2023a) as indicative of species level divergence. The tree-based approaches were the single and multi-rate Poisson tree process (PTP and mPTP) methods (Zhang et al. 2013; Kapli et al. 2017). The ML tree was trimmed to include only members of the *C. nebulosus* complex with one sequence of *C. jeyporensis* as an outgroup. Analyses were conducted using web servers (https://mptp.h-its.org/#/tree; https://species.h-its.org/) and used default settings with the outgroup dropped and a P-value of 0.001 for PTP.

### Divergence dating

We used a trimmed alignment that included only a single lineage per species of the *triedrus* clade for divergence dating in BEAST 2.7.7 (Bouckaert et al. 2019). We used a single partition, BEAST model test, Yule speciation prior, and a secondary calibration point for the most recent common ancestor (MRCA) of the *triedrus* group from Agarwal and Karanth (2015) of 32 (36–27) million years ago (mya) with a normal prior (mean 32, sigma 2.5). The analysis was run for 20 × 10^6^ generations, sampling every 2,000, with the first 25% discarded as burn-in. Convergence was determined based on ESS values (>>200), and a Maximum Clade Credibility Tree built in TreeAnnotator 2.7.7.

### Morphological data

We examined the type series at the Natural History Museum, London, UK (**NHMUK**, although physical tags bear the earlier acronym **BMNH** numbers), in addition to topotypical specimens in the collection of the National Centre for Biological Sciences, Bangalore (**NRC-AA**) from the vicinity of Galikonda, near Araku, Visakhapatnam District, Andhra Pradesh collected between elevations of 300–1200 m. Morphological data was collected from a total of 18 specimens of *C. nebulosus* and two specimens of the new species. We follow the methods of Agarwal et al. (2023a) and Bhattarai et al. (2025a, 2025b) for morphological data. We recorded colour patterns from photographs of live specimens and morphological data using a ZEISS Stereo Discovery V8 dissecting microscope on the left side of the body whenever possible, with bilateral scale counts taken on both sides of each specimen. VPC examined the syntypes of *C. nebulosus* and AK all other specimens. As the types of *C. nebulosus* are extremely faded, colour pattern is reported based on topotypical specimens, and from the type series only where pattern was discernible. The following measurements were taken with a Mitutoyo digital caliper (to the nearest 0.1 mm): snout vent length (**SVL**, from tip of the snout to cloacal opening); tail length (**TL**, from cloaca to tail tip); tail width (**TW**, measured at tail base); lower arm length (**LAL**, from elbow to distal end of wrist; measured by flexing elbow at 90° wherever needed); crus length (**CL**, from knee to heel; measured by flexing knee at 90° wherever needed); axilla to groin length (**AGL**, from posterior margin of forelimb insertion to anterior margin of hindlimb insertion on the body); body height (**BH**, maximum height of body measured at midbody); body width (**BW**, maximum width of body measured at midbody); head length (**HL**, distance from the retroarticular process to the snout tip); head width (**HW**, maximum width of head, measured just behind the eyes); head height (**HH**, maximum height of head measured at the level of the eye); eye diameter (**ED**, greatest horizontal diameter of eye); eye to ear distance (**EE**, distance from anterior edge of ear opening to posterior margin of eye); eye to snout distance (**ES**, distance between anterior margin of eye and tip of snout); eye to nares distance (**EN**, distance between anterior margin of eye and posterior edge of nostril); internarial distance (**IN**, distance between nares measured dorsally from their internal margins); interorbital distance (**IO**, shortest distance between left and right supraciliary scale rows in front of orbit); and ear length (**EL**, maximum length of ear opening).

The following meristic data were recorded for all specimens: number of internasals (**INS**, number of scales behind rostral and between supranasals); number of supralabials (**SL**), and infralabials (**IL**), from rostral and mental, respectively, to posterior-most enlarged scale at angle of the jaw; supralabials at midorbital position (**SL M**), and infralabials at midorbital position (**IL M**), from rostral and mental, respectively, to below the middle of the eye; paravertebral tubercles (**PVT**, number of enlarged tubercles between limb insertions counted in a straight line immediately left or right of the vertebral column); dorsal tubercle rows (**DTR**, number of longitudinal rows of enlarged tubercles around the body counted at midbody); mid ventral scale rows (**MVSR**, enlarged ventral scales counted across the body midbody between lowest rows of much smaller dorsal granular scales); ventral scales 1 (**VS1**, counted on midbody ventral between forelimb and hindlimb insertions); ventral scales 2 (**VS2**, counted from the mental to anterior border of the cloacal opening); distal subdigital lamellae counted from digital inflection at first phalanx to the claw, excluding the large scale on inflection and including the claw sheath on manus: digit 1 (**DLAMF1**), digit 4 (**DLAMF4**), on pes: digit 1 (**DLAMT1**), digit 4 (**DLAMT4**), and digit 5 (**DLAMT5**); basal subdigital lamellae, counted from digital inflection at first phalanx (including the large scale on inflection) to the base of the digits including all scales that are wider than high; on manus: digit 1 (**BLAMF1**), digit 4 (**BLAMF4**), on pes: digit 1 (**BLAMT1**), digit 4 (**BLAMT4**), and digit 5 (**BLAMT5**); total lamellae (**TLAMF1**, **TLAMF4**, **TLAMT1**, **TLAMT4**, and **TLAMT5** are sum of respective basal and distal lamellae for all digits); and post cloacal tubercles (**PCT**, number of post cloacal tubercles on either side of the tail base).

## Results

### Phylogenetic relationships

**Figure 2.**
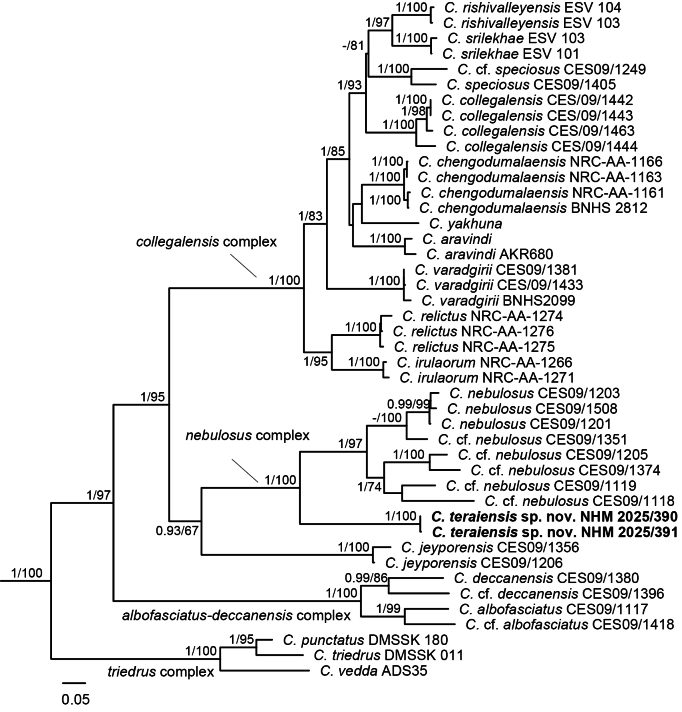
Maximum likelihood phylogeny of Cyrtodactylus (Geckoella). Posterior probability (≥ 0.90)/ ML bootstrap support (≥ 65) for RaXML shown at nodes; outgroups not shown.

We recover a well-supported, monophyletic *Geckoella* clade (Fig. 2). A basal split separates the *Cyrtodactylus triedrus* complex, and the remaining species fall within a wet zone clade (*C. albofasciatus* + *C. deccanensis* complexes) and a dry zone clade. Within the latter clade, the *C. collegalensis* complex is sister to the *C. jeyporensis* + the *C. nebulosus* complex. The new species from Nepal forms a strongly supported sister taxon to the entire *C. nebulosus* complex from India. The new species is 17.3% divergent from *C. nebulosus*, 15.7–18.1% from other members of the *C. nebulosus* complex, and > 21.0% from other *Geckoella* (Table 2). All members of the *C. nebulosus* complex and *C. jeyporensis* have a 6-nt deletion between positions 661–666 in the ND2 alignment; with an additional 3-nt deletion between 960–962 in all Indian members of the *C. nebulosus* complex (absent from the Nepal population).

### Species delimitation and divergence dates

Employing the 8.7% uncorrected ND2 sequence divergence cut-off recovered five species within the *nebulosus* complex (Fig. 2). The tree-based methods both recovered seven species, *C. nebulosus*, the Nepal population, and *C. cf. nebulosus* 1–5 (Fig. 2).

**Figure 3.**
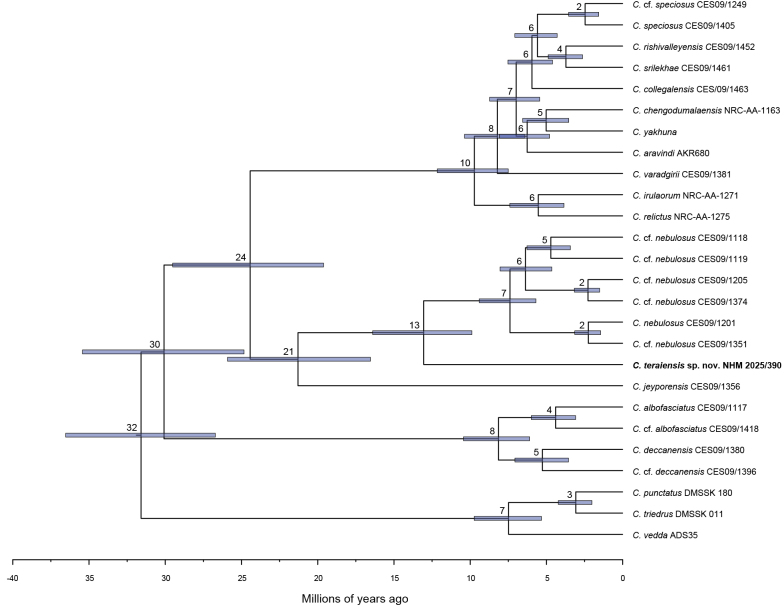
Time tree for Cyrtodactylus (Geckoella). Numbers at nodes are median node age and bars 95% HPD.

The wet zone clade was estimated to have diverged from the dry zone clade 30 (35–25) mya during the late Eocene–early Oligocene. The *C. collegalensis* complex split from the *C. jeyporensis* + *C. nebulosus* clade 24 (30–20) mya in the late Oligocene-early Miocene, but began diversifying 10 (12–8) mya in the Late Miocene (Fig. 3). *Cyrtodactylus jeyporensis* split from the MRCA of the *C. nebulosus* complex 21 (26–17) mya in the early Miocene and the Nepal population of *C. nebulosus* diverged from Indian members 13 (16–10) mya in the mid Miocene; diversification within Indian lineages began approximately seven (9–6) mya in the late Miocene.

### Systematics

#### Cyrtodactylus (Geckoella) nebulosus (Beddome, 1870)

##### Gymnodactylus nebulosus

Taxon classificationAnimaliaSquamataPhyllodactylidae

Beddome, 1870

35BD11F2-5F3F-52CC-B59F-D711FF0D4693

Fig. 4; Tables 3, 4

###### Type material examined.

***Lectotype***. • (Designated herein) NHMUK 1946.9.7.51, female, SVL 33.2 mm, from “Golcondah hills near Vizagapatam [= Visakhapatnam, Andhra Pradesh]… at 2,000–3,500 feet [= ~600–1100 m] elevation”; collected by coll. R.H. Beddome. ***Paralectotypes***. • NHMUK 1946.9.7.52–54 (one subadult female and two unsexed juveniles); same collection data as lectotype.

**Figure 4.**
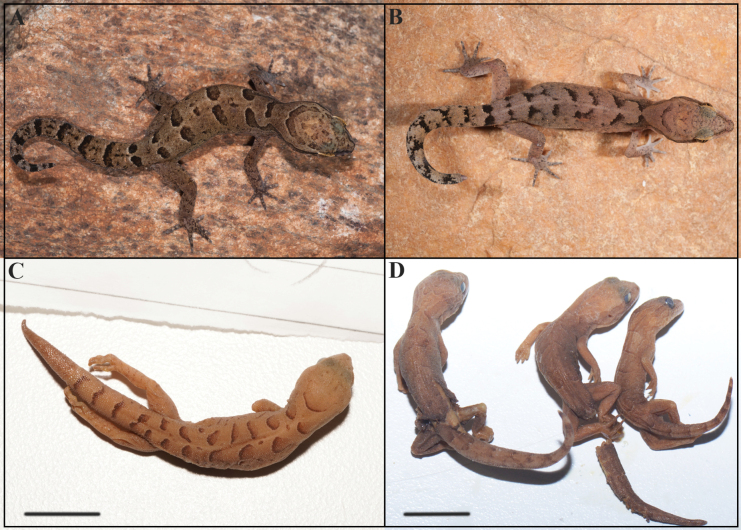
Dorsal view of Cyrtodactylus (Geckoella) nebulosus. **A**. Topotype in life (adult male, NRC-AA-9628); **B**. Topotype in life (adult female, NRC-AA-9619); **C**. Lectotype (female, NHMUK 1946.9.7.51); **D**. Paralectotypes (NHMUK 1946.9.7.52–54). Photographs by IA (**A, B**), and VPC (**C, D**). Scale bars: 10 mm.

**Table 3.** Mensural data (in mm) of Cyrtodactylus (Geckoella) nebulosus. Abbreviations are listed in Materials and Methods except for: M = male, F = female, Asterisk (*) = tail incomplete; L & R = left and right, ? = could not be determined.

Type	Lectotype	Paralectotypes			Referred material (topotypes)													
Specimen No.	NHMUK 1946.9.7.51	NHMUK 1946.9.7.52	NHMUK 1946.9.7.53	NHMUK 1946.9.7.54	NRC-AA-9616	NRC-AA-9617	NRC-AA-9618	NRC-AA-9619	NRC-AA-9620	NRC-AA-9621	NRC-AA-9622	NRC-AA-9623	NRC-AA-9624	NRC-AA-9625	NRC-AA-9626	NRC-AA-9627	NRC-AA-9628	NRC-AA-9629
Sex	F	F	?	?	F	F	M	F	F	F	F	F	F	F	M	F	M	M
SVL	33.2	28.5	27.8	23.2	42.3	43.8	37.5	46.1	47.9	45.6	40.1	37.7	42.7	44.0	47.5	52.1	52.2	47.3
TL	23.2	21.8	19.2*	15.3	35.2	36.5	21.5*	31.6	20.4*	40.1	33.3	31.3	31.8	38.0	28.3	11.0*	40.1	20.6*
TW	3.5	2.7	2.2	1.6	3.6	3.4	3.5	3.6	4.5	4.0	3.5	2.9	3.7	4.1	5.3	4.5	4.7	5.1
LAL	3.7	3.2	3.1	2.6	5.5	5.8	5.0	6.2	6.5	6.0	5.4	5.5	5.6	5.8	6.4	7.2	7.6	7.1
CL	5.7	5.0	4.7	4.2	7.3	7.4	6.3	7.6	8.2	7.7	6.4	6.2	6.8	7.7	7.9	8.8	9.2	8.2
AGL	14.1	13.9	13.8	10.8	17.7	18.1	16.2	20.3	19.5	20.3	16.4	16.4	18.5	19.2	21.1	25.1	26.6	21.5
BH	4.3	4.0	3.8	3.2	4.2	4.0	3.6	5.2	6.5	4.6	4.7	4.2	3.7	5.9	6.2	5.9	5.0	6.2
BW	5.7	4.8	4.7	3.8	7.0	7.1	6.8	7.7	9.8	8.2	7.4	5.6	8.2	9.6	9.7	11.1	10.2	10.2
HL	9.1	8.2	7.6	6.2	10.9	11.5	9.4	11.5	11.2	11.2	10.8	10.0	10.9	10.2	12.7	12.7	12.8	11.8
HW	6.8	6.1	6.1	5.2	7.7	7.7	6.3	8.3	8.3	7.8	7.6	6.7	7.3	7.7	8.8	9.3	9.7	8.9
HD	4.3	4.1	3.8	3.6	4.6	4.6	4.2	4.9	5.5	5.1	4.3	4.1	5.4	5.1	5.7	5.8	6.1	5.6
ED	2.2	1.8	1.8	1.7	2.4	2.8	2.1	2.5	2.8	2.5	2.5	2.4	2.6	2.4	3.1	3.1	3.1	3.1
EE	3.0	2.7	2.4	2.0	3.3	3.6	3.0	3.7	4.1	3.6	3.3	3.1	3.4	3.7	3.8	4.2	4.1	3.5
ES	3.8	3.2	3.2	2.8	4.8	4.8	4.2	5.3	5.1	4.9	4.7	4.0	4.9	5.0	5.3	5.7	6.0	5.1
EN	2.9	2.3	2.3	2.1	3.5	3.6	3.1	3.7	3.7	3.6	3.6	2.9	3.5	3.7	3.7	4.2	4.2	3.8
IN	1.4	1.1	1.2	1.1	1.2	1.5	1.1	1.3	1.5	1.5	1.3	1.2	1.5	1.4	1.6	1.5	1.7	1.4
IO	2.9	2.4	2.5	2.4	2.1	2.1	1.9	2.0	2.7	2.4	2.3	1.7	2.0	2.5	2.8	2.7	2.8	2.5
EL	0.8	0.6	0.8	0.7	0.8	0.9	0.9	1.1	0.8	1.2	1.1	0.8	1.1	1.0	1.3	1.4	1.4	1.7

**Table 4.** Meristic data of Cyrtodactylus (Geckoella) nebulosus. Abbreviations are listed in Materials and methods except for: M = male, F = female, Asterisk (*) = data incomplete; L&R = left and right, / = data unavailable, ? = could not be determined.

Type	Lectotype		Paralectotypes		Referred material (topotypes)													
Specimen No.	NHMUK 1946.9.7.51	NHMUK 1946.9.7.52	NHMUK 1946.9.7.53	NHMUK 1946.9.7.54	NRC-AA-9616	NRC-AA-9617	NRC-AA-9618	NRC-AA-9619	NRC-AA-9620	NRC-AA-9621	NRC-AA-9622	NRC-AA-9623	NRC-AA-9624	NRC-AA-9625	NRC-AA-9626	NRC-AA-9627	NRC-AA-9628	NRC-AA-9629
Sex	F	F	?	?	F	F	M	F	F	F	F	F	F	F	M	F	M	M
INS	1	2	2	1	1	1	1	1	1	2	2	2	2	2	3	1	1	3
SL L&R	9&10	10&10	10&10	13&12	11&10	12&12	11&11	14&13	12&12	10&10	13&12	11&10	11&11	10&11	10&10	11&12	12&10	11&11
IL L&R	8&7	9&9	8&9	9&8	8&8	9&8	8&8	9&10	9&9	8&8	10&9	10&9	8&9	8&8	8&9	9&8	9&9	9&9
SL M L&R	6&7	7&7	7&7	8&7	7&7	7&7	7&7	7&7	7&6	6&7	7&7	7&6	8&8	6&6	6&6	7&7	8&6	6&6
IL M L&R	6&5	6&6	6&6	6&6	5&5	6&5	5&6	6&6	5&5	5&6	6&6	6&5	6&6	5&5	5&5	5&5	6&6	6&5
PVT L&R	33&33	32*	31	/	27&27	27&29	25&25	30&29	27&30	30&26	27&27	28&28	28&28	27&26	31&32	27&28	32&32	28&27
DTR	13	14	11	/	12	12	12	12	12	12	13	14	13	12	14	12	12	12
MVSR	35	37	42	33	/	38	41	40	34	38	40	/	40	38	38	40	37	37
VS1	55	/	57	62	60	57	60	62	57	60	60	/	55	52	55	58	60	56
VS2	/	/	/	/	163	159	165	169	172	175	164	/	150	140	150	161	157	153
DLAMF1 L&R	6&6	7&6	7&7	7&6	7&7	7&7	7&6	7&7	7&7	7&7	7&7	7&7	7&7	7&7	7&7	7&6	7&7	6&6
BLAMF1 L&R	5&5	3&4	4&4	5&5	4&4	3&4	3&5	5&4	4&4	4&4	5&5	4&4	4&4	4&4	4&4	4&5	4&4	4&4
DLAMF4 L&R	8&8	8&8	7&8	7&8	7&7	8&8	8&8	8&2*	9&1*	6*&8	8&7	8&8	7&8	7&8	8&8	8&8	7&7	7&7
BLAMF4 L&R	6&6	5&5	5&5	5&6	4&4	5&5	5&5	5&5	5&5	6&6	5&5	6&5	5&5	6&5	4&4	6&6	5&5	5&4
DLAMT1 L&R	7&7	7&7	7&8	7&7	7&7	9&8	8&8	8&8	8&8	8&8	8&8	8&8	8&7	8&8	7&7	7&7	6&7	7&7
BLAMT1 L&R	3&4	4&4	5&4	4&4	4&6	3&3	3&3	3&3	4&4	4&5	3&3	4&4	3&3	3&3	4&4	5&5	4&3	3&3
DLAMT4 L&R	9&10	9&9	9&9	11&9	9&7	9&9	9&9	9&9	9&9	9&9	9&9	9&9	8&9	9&9	9&9	9&9	8&8	9&9
BLAMT4 L&R	8&7	7&7	7&7	7&7	8&7	8&8	8&7	7&7	7&7	7&7	7&7	7&7	6&6	7&7	7&7	8&7	6&6	5&5
DLAMT5 L&R	12&11	10&9	10&10	11&11	10&10	10&9	10&10	9&9	11&10	10&10	10&10	9&10	10&10	10&10	10&11	9&9	8&9	9&10
BLAMT5 L&R	6&6	7&7	6&6	6&6	5&6	5&6	7&6	6&6	6&5	6&6	7&7	6&5	5&5	6&6	6&6	6&6	5&6	5&5
TLAMF1 L&R	11&11	10&10	11&11	12&11	11&11	10&11	10&11	12&11	11&11	11&11	12&12	11&11	11&11	11&11	11&11	11&11	11&11	10&10
TLAMF4 L&R	14&14	13&13	13&14	12&14	11&11	13&13	13&13	13&7*	14&6*	12&14	13&12	14&13	12&13	13&13	12&12	14&14	12&12	12&11
TLAMT1 L&R	10&11	11&11	12&12	11&11	11&13	12&11	11&11	11&11	12&12	12&13	11&11	12&12	11&10	11&11	11&11	12&12	10&10	10&10
TLAMT4 L&R	17&17	16&16	16&16	18&16	17&14	17&17	17&16	16&16	16&16	16&16	16&16	16&16	14&15	16&16	16&16	17&16	14&14	14&14
TLAMT5 L&R	18&17	17&16	16&16	17&17	15&16	15&15	17&16	15&15	17&15	16&16	17&17	15&15	15&15	16&16	16&17	15&15	13&15	14&15
PCT L&R	2&2	2&2	1&1	2&2	2&2	3&3	4&3	3&3	2&2	2&2	2&2	2&2	2&2	2&2	2&2	2&2	2&2	3&4

###### Additional material.

• NRC-AA-9618 (CES09/1510), NRC-AA-9626 (CES09/1518), NRC-AA-9628 (CES09/1202), NRC-AA-9629 (CES09/1203), adult males and NRC-AA-9616 (CES09/1508), NRC-AA-9617 (CES09/1509), NRC-AA-9619 (CES09/1511), NRC-AA-9620 (CES09/1512), NRC-AA-9621 (CES09/1513), NRC-AA-9622 (CES09/1514), NRC-AA-9623 (CES09/1505), NRC-AA-9624 (CES09/15016), NRC-AA-9625 (CES09/1517), NRC-AA-9627 (CES09/1201), adult females; collected from the road up to Araku Valley (18.21355°N, 83.05556°E – 18.27687°N, 82.98932°E; ca 310–1170 m a.s.l.), Alluri Sitharama Raju District, Andhra Pradesh State, India; by Aniruddha Datta-Roy, Aparna Lajmi, V. Deepak and Ishan Agarwal on 3 March 2014.

###### Diagnosis.

A small-sized *Cyrtodactylus*, snout to vent length up to 52.2 mm. Dorsal pholidosis heterogeneous; smooth granular scales intermixed with more or less regularly arranged rows of enlarged, feebly keeled, blunt to weakly conical tubercles; ventrolateral fold absent on lower flank; 12–14 rows of dorsal tubercles at midbody (rarely 11, *n* = 1/17), 25–33 tubercles in paravertebral rows; ventral scales subequal from chest to vent, smooth, subcircular, and subimbricate with rounded end; 37–42 ventral scales across belly at midbody (rarely 33–35, *n* = 3/16); 52–62 longitudinal scales between axilla to groin; 150–175 longitudinal scales from mental to cloaca (rarely 140, *n* = 1/13); subdigital scansors smooth, unnotched, and mostly entire; 10–13 lamellae under digit I of manus and nine or ten lamellae under digit I of pes, 11–14 lamellae under digit IV of manus and 14–17 lamellae under digit IV of pes; femoral and/or precloacal pores absent; enlarged precloacal and/ or femoral scales absent, no precloacal groove; scales on non-regenerated tail dorsum homogeneous, composed of large, flat, smooth, subcircular, and imbricate scales which becomes slightly larger towards the lateral aspect, largest on ventral side but not forming median row of transversely enlarged subcaudal scales. Ground colour brown with 5–8 pairs of dark brown spots or streaks from neck to hindlimb insertions plus one on tail base; head mottled with numerous dark blotches; post-occipital collar extends to posterior margin of eye; pre-orbital streaks do not meet in internasal region; post-orbital streak extends onto back and is confluent with first or first and second dorsal markings; original tail with 6–9 alternating dark and lighter bands.

###### Note.

The relatively specific type locality in the original description of *C. nebulosus* and comparison against the type material allowed us to assign our topotypical specimens to this species. Additionally, the original description gives the size as 2 1/4 inches [= 57 mm] and 34 scales across the belly which matches well with the specimen selected as the lectotype (total length, SVL + TL = 56.4 mm and 35 MVSR). The lectotype is also the largest specimen (although it is likely not fully adult) and in the best condition among the syntype series.

##### Cyrtodactylus (Geckoella) teraiensis sp. nov.

Taxon classificationAnimaliaSquamataGekkonidae

B5F9010E-170D-5532-B27C-44ECE8855462

https://zoobank.org/3C1A0B0E-617D-4929-AA9B-A1E791B1E9EA

Figs 5, 6, 7, 8, 9

###### Type material.

***Holotype***. • NHM 2025/390 (BG-81266), adult male, collected from Belbari Chisang Collaborative Forest (26.67993°N, 87.4743°E; ca 188 m a.s.l.), Bhaunne, Morang District, Koshi Province, Nepal; by Santosh Bhattarai and Bivek Gautam on 27^th^ April 2025. ***Paratype***. • NHM 2025/391 (BG-81277), adult male, Belbari Chisang Collaborative Forest (26.684517°N, 87.472117°E; ca. 169 m a.s.l.), Bhaunne, bears the same locality and collection data as holotype.

###### Diagnosis.

A small-sized *Cyrtodactylus*, snout to vent length up to 45.5 mm. Dorsal pholidosis heterogeneous; smooth granular scales intermixed with more or less regularly arranged rows of enlarged, feebly keeled, blunt to weakly conical tubercles; ventrolateral fold absent on lower flank; 16–18 rows of dorsal tubercles at midbody, 29–31 tubercles in paravertebral rows; ventral scales subequal from chest to vent, smooth, subcircular, and subimbricate with rounded end; 30–32 ventral scales across belly at midbody, 50–54 longitudinal scales between axilla to groin, 150–157 longitudinal scales from mental to cloaca; subdigital scansors smooth, unnotched, and mostly entire; 8–10 lamellae under digit I of manus and nine or 10 lamellae under digit I of pes, 12 or 13 lamellae under digit IV of manus and 15–17 lamellae under digit IV of pes; femoral and/or precloacal pores absent; enlarged precloacal and/ or femoral scales absent, no precloacal groove; scales on non-regenerated tail dorsum homogeneous, composed of large, flat, smooth, subcircular, and imbricate scales which become slightly larger towards the lateral aspect, largest on ventral side but not forming median row of transversely enlarged subcaudal scales. Ground colour khaki with four or five pairs of dark brown spots from neck to hindlimb insertions plus one on tail base; head with dark blotches on snout-tip, occipital, and interorbital region; post-occipital collar not in contact with post-orbital streak; pre-orbital streaks meeting in internasal region; original tail with eight or nine alternating dark and lighter bands.

###### Comparison with Cyrtodactylus (Geckoella) nebulosus.

The small body size and original tail shorter than SVL easily differentiate the new species from all other *Cyrtodactylus* and its dorsal pholidosis from all Cyrtodactylus (Geckoella) apart from *C. nebulosus*.

Cyrtodactylus (Geckoella) teraiensis sp. nov. can be differentiated from *C. nebulosus* by having 16–18 rows of dorsal tubercles at midbody (vs 12–14 rows of dorsal tubercles at midbody in *C. nebulosus*); 30–32 ventral scales across belly at midbody (vs 37–42 ventral scales across belly at midbody; rarely 33–35, *n* = 3/16); four or five paired spots between neck and hindlimb insertions alternating with two or three much smaller paired spots (vs 4–7 dark dorsal markings between neck and hindlimb insertions that vary from irregular paired blotches to cross-bars alternating with 1–3 small paired spots/ cross-bars); post-occipital collar does not extend up to eye (vs post-occipital collar extends to posterior margin of eye); post-orbital streak terminates before tympanum (vs post-orbital streak extends onto back and is confluent with first or first and second dorsal markings); pre-orbital streaks meet on internasals (vs pre-orbital streaks do not meet on internasals).

###### Description of the holotype.

**Figure 5.**
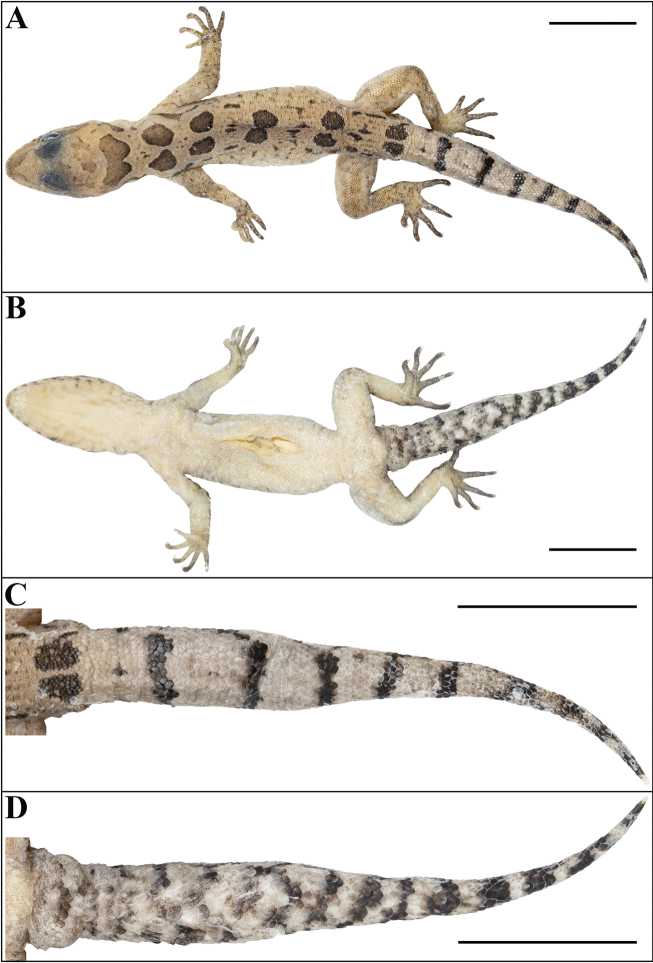
Cyrtodactylus (Geckoella) teraiensis sp. nov. (holotype, NHM 2025/390). **A**. Dorsal view of body; **B**. Ventral view of body; **C**. Dorsal view of tail; **D**. Ventral view of tail. Photographs by AK. Scale bars: 10 mm.

**Figure 6.**
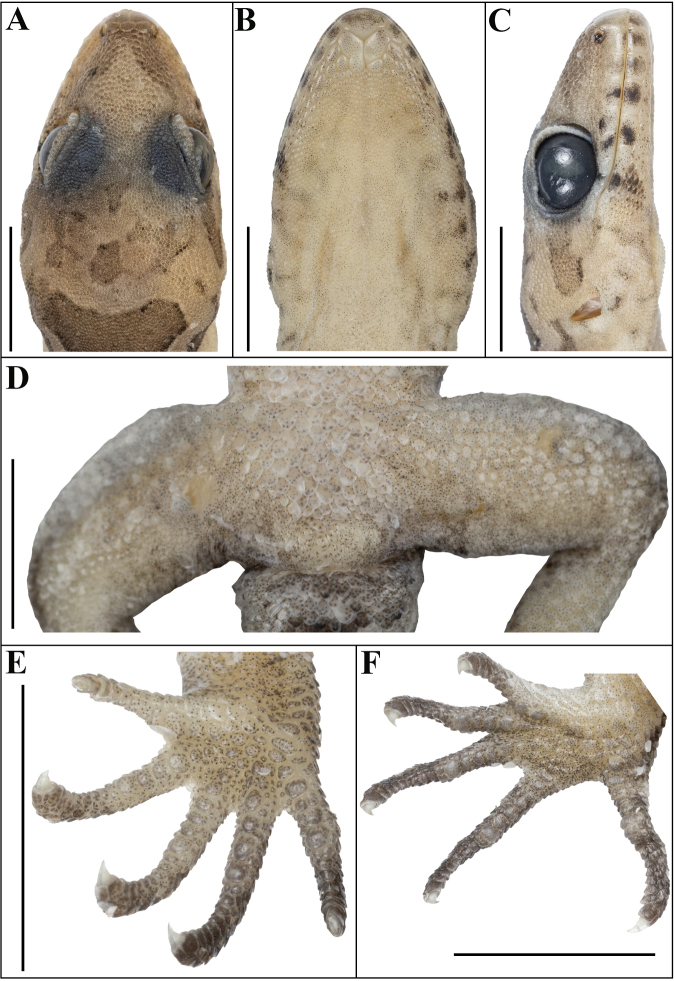
Holotype of Cyrtodactylus (Geckoella) teraiensis sp. nov. (holotype, NHM 2025/390). **A**. Dorsal view of head; **B**. Ventral view of head; **C**. Lateral view of head on right; **D**. View of femoral region; **E**. Ventral view of left manus; **F**. Ventral view of left pes. Photographs by AK. Scale bars: 5 mm.

Adult male in good state of preservation except body and tail slightly bent towards left and a 9.4-mm vertical incision in the sternal region for liver tissue collection (Fig. 5A–D). SVL 44.6 mm, head short (HL/SVL 0.26), wide (HW/HL 0.69), not strongly depressed (HD/HL 0.43), as broad as body (HW/BW 1.01), and distinct from neck (Fig. 6A). Loreal region slightly inflated, canthus rostralis not prominent. Snout marginally less than half head length (ES/HL 0.42), slightly less than twice eye diameter (ES/ED 1.85); scales on snout, canthus rostralis, and loreal region large, hexagonal to subcircular, smooth and flattened; much larger than granular scales on forehead and interorbital region; occipital and temporal region with heterogenous scalation consisting of much smaller, smooth granular scales intermixed with slightly enlarged, smooth to feebly keeled, round tubercles (Fig. 6A, C). Eye small (ED/HL 0.23); pupil, dilated, vertical with crenate margins; supraciliaries short, larger anteriorly, not elongate; 12 interorbital scale rows across narrowest point of frontal; 35 scale rows between left and right supraciliaries at mid-orbit (Fig. 6A, C). Ear-opening oval, small (EL/HL 0.10); eye to ear distance slightly larger than eye diameter (EE/ED 1.35). Rostral ~ 1.5 × wider (2.3 mm) than deep (1.6 mm), incompletely divided dorsally by weakly developed rostral groove for half of its height; a single enlarged supranasal on each side, more than twice the size of the postnasals, separated from each other by a single enlarged internasal on the snout; three subequal postnasals, much smaller than supranasals; rostral in contact with nostril, supralabial I, supranasal, and internasal on either side; nostrils rounded, directed somewhat outwards, covering most of the nasal scale; surrounded on either side by supralabial I, rostral, supranasals, and postnasals; a single row of smaller scales separate the orbit from the supralabials (Fig. 6C). Mental enlarged, triangular, wider (2.1 mm) than long (1.4 mm); two pairs of postmentals; inner pair in strong contact with each other, approx. pentangular and slightly smaller in length (1.8 mm) than mental; bordered by mental, infralabial I, outer postmental and seven enlarged chin shields below; outer postmentals separated from each other by left inner postmentals, approx. rectangular and half the size (0.90 mm) of inner pair; bordered by inner postmentals, infralabials I and II, and three enlarged chin shields on either side; chin shields bordering postmentals and infralabials flat, smooth, smaller than outermost postmentals; rest flattened, even smaller, smooth; two or three rows of enlarged elongated scales separating gular scales from infralabials (Fig. 6B). Twelve supralabials up to angle of jaw on either side, and seven at midorbital position on left and six on right side; nine infralabials up to angle of jaw on the left and eight on the right side, and six infralabials at midorbital position on left and five on right side (Fig. 6C).

**Figure 7.**
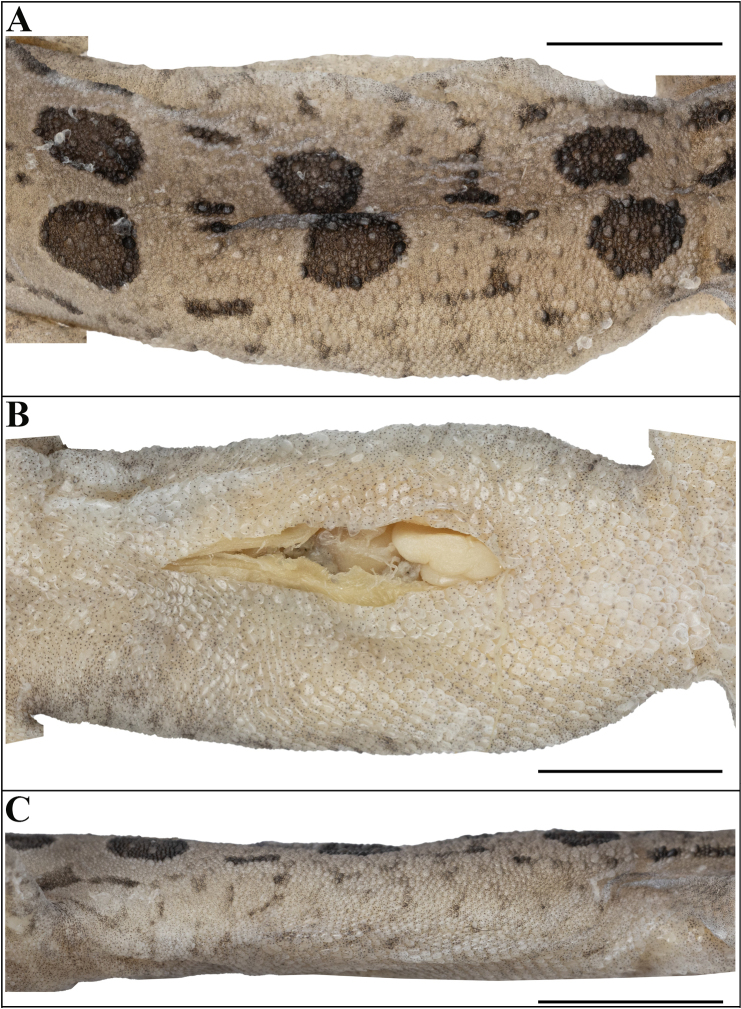
Holotype of Cyrtodactylus (Geckoella) teraiensis sp. nov. (holotype, NHM 2025/390). **A**. Dorsal view of midbody; **B**. Ventral view of midbody; **C**. Lateral view of midbody on right. Photographs by AK. Scale bars: 5 mm.

Body relatively slender (BW/AGL 0.47), trunk less than half of SVL (AGL/SVL 0.39) without ventrolateral folds (Fig. 7A–C). Dorsal pholidosis on trunk heterogeneous; smooth granular scales intermixed with more or less regularly arranged rows of enlarged, feebly keeled, blunt to weakly conical tubercles; granular scales gradually increasing in size towards each flank, largest on mid-flank; granular scales on occiput slightly smaller than paravertebral granular scales; enlarged tubercles in ~ 16 longitudinal rows at midbody; 29 tubercles in paravertebral rows (Fig. 7A). Ventral scales much larger than granular scales on dorsum, subequal from chest to vent, smooth, subcircular, and subimbricate with rounded end; no enlarged precloacal or femoral scales, no precloacal or femoral pores and no precloacal groove (Figs 6D, 7B).

Scales on palm and soles granular, smooth, rounded; scales on dorsal aspects of forelimbs heterogeneous in shape and size; mixture of small, granules similar to dorsum and many smooth flattened and imbricate scales which are much larger than granules on the body dorsum, largest on anterior aspect of the hands; lateral and ventral aspects of forelimbs with small granular scales; scales on dorsal aspects of forelimbs heterogeneous, smaller scales intermixed with sparsely arranged tubercles (Fig. 5A, B). Fore-limbs and hind-limbs long, slender (LAL/SVL 0.14; CL/SVL 0.17); digits short, slender, with a strong, recurved claw, moderately inflected, distal portions laterally compressed. Series of unpaired lamellae on basal portion of digits except one or two which on some digits which are paired, separated from narrower distal lamellae by a single large lamella at the inflection, unpaired except one or two which are divided; basal lamellae series: (3-4-5-4-5 right manus, 2-5-6-8-6 right pes), (3-4-4-5-4 left manus, Fig. 6E; 2-6-6-8-5 left pes, Fig. 6F); distal lamellae series: (7-7-9-8-7 right manus, 7-8-10-9-10 right pes), (7-7-9-8-7 left manus, Fig. 6E; 7-8-10-9-10 left pes, Fig. 6F). Relative length of digits (measurements in mm in parentheses): IV (3.4) > III (3.2) > II (2.7) > V (2.4) > I (2.1) (right manus); IV (4.4) > III (4.0) = V (4.0) > II (3.1) > I (2.4) (right pes).

Tail original, circular in cross section with indistinct median dorsal furrow, relatively thick, tapering gradually to tip, unsegmented, slightly shorter than snout-vent length (TL/SVL 0.80). Scales on dorsal aspect of tail base similar to body dorsum; scales on dorsal aspect of tail large, flat, subcircular, smooth, and imbricate, becoming slightly larger towards the lateral aspect, largest on ventral side, but not forming median row of transversely enlarged subcaudal scales (Fig. 5C, D). Two small, smooth, subequal, conical postcloacal spurs on either side of tail base; prominent hemipenial swelling, a small flap of skin covering cloacal aperture. Tail slightly constricted at the base (Fig. 5D).

###### Additional mensural and meristic data.

All measurements in mm; abbreviations as listed in Materials and Methods except for L&R = left and right. Mensural data: TL = 35.9, TW = 4.8, LAL = 6.3, CL = 7.9, AGL = 17.4, BH = 4.5, BW = 8.2, HL = 12.0, HW = 8.3, HD = 5.2, ED = 2.8, EE = 3.8, ES = 5.1, EN = 3.7, IN = 1.9, IO = 2.8, and EL = 1.3; meristic data: INS = 1, TLAMF1 (L&R) = 10&10, TLAMF4 (L&R) = 13&12, TLAMT1 (L&R) = 9&9, TLAMT4 (L&R) = 17&17, and TLAMT5 (L&R) = 15&16.

###### Colouration in life (Fig. 8A).

Dorsal ground colour khaki, four pairs of dark brown spots from behind occiput to hindlimb insertions and one pair above tail base, separated mid-vertebrally and not extending onto lateral edge of body; approx. subequal with largest between forelimb insertions and smallest on tail base; spots with a fine cream border; lighter interspaces between collar and 1^st^ pair of spots and between 1^st^ and 2^nd^ pair of spots approx. equal to thickness of spots, lighter interspace between other pairs of spots ~2 × spot thickness and with small black paired spots; scattered black spots on dorsum and flanks. Tail dorsum sightly lighter than body dorsum with a faint yellow hue; ~9 dark crossbars on tail. Dorsum of limbs and digits faded compared to body with indistinct grey reticulations and a few scattered light blotches. Post-occipital collar formed of two fused brown spots forming a B-shaped marking that is approx. equal to width of spots on dorsum, posteriorly darker with a fine light border, not in contact with post-orbital streak. Head dorsum slightly duller than trunk with few scattered dark brown spots, and five dark brown irregular blotches — an approx. V-shaped marking on snout-tip formed by pre-ocular streaks which meet in internasal region, a narrow streak between anterior margins of orbit, a broken broader streak at centre and posterior edge of orbit with a subcircular spot posterior to posterior margin of orbit, a parietal central subcircular blotch flanked by two elongate markings. Brille similar in colour to crown; postocular streak between posterior edge of eye to before tympanum; fine dark streak continuing laterally below collar and terminating before first pair of dorsal spots on neck. Labials with few dark streaks, a few unmarked scales finely spotted with black. Ventral aspects dirty white with numerous short streaks and spots on infralabials and gular region; ventral aspect of tail with thick dark reticulations and light markings.

**Figure 8.**
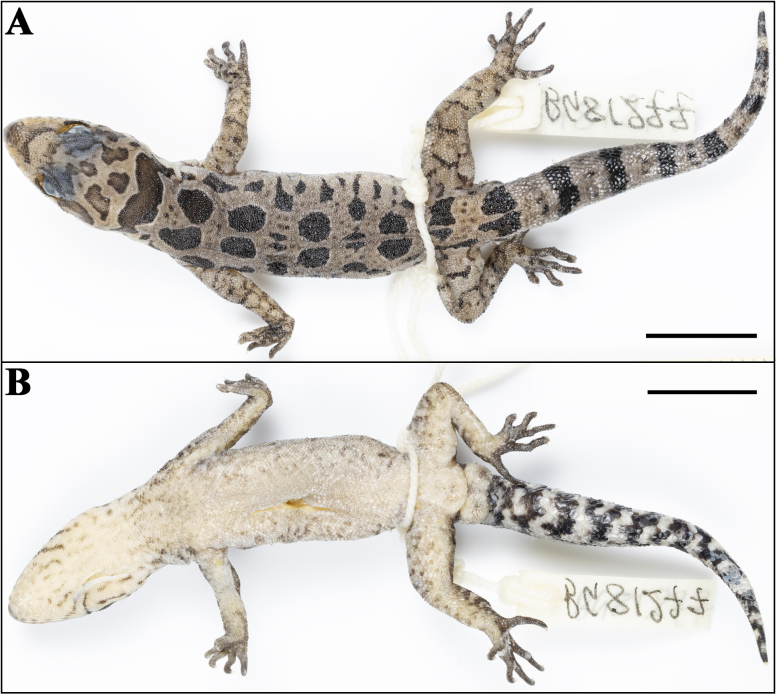
Paratype (adult male, NHM 2025/391) of Cyrtodactylus (Geckoella) teraiensis sp. nov. **A**. Dorsal view; **B**. Ventral view. Photographs by AK. Scale bar: 10 mm.

###### Variation and additional information from paratype.

**Figure 9.**
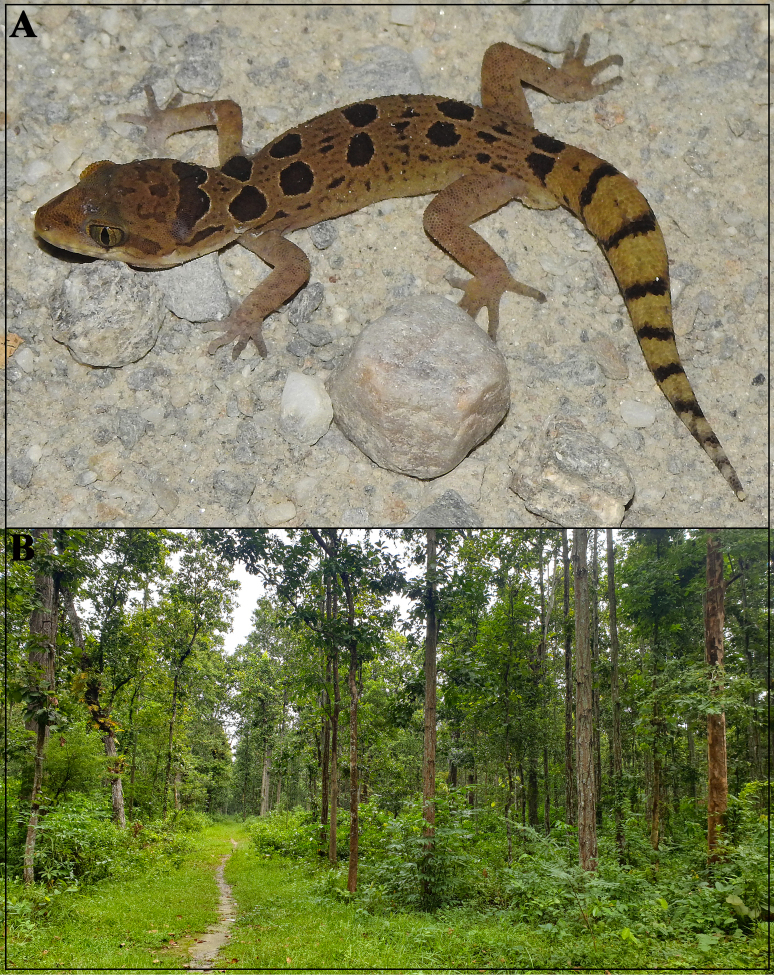
Photo of: **A**. Holotype of Cyrtodactylus (Geckoella) teraiensis sp. nov. in life; **B**. Habitat from where the new species was collected. Photographs by BG.

The adult male paratype NHM 2025/391 (BG-81277) resembles the holotype except as follows: inner postmentals bordered by mental, infralabial I, and outer postmental; additionally, bordered by two enlarged chin shields. Outer postmentals bordered by inner pair and infralabial I and II; additionally, bordered by three smaller chin shields on left and four on right side. Mensural data: SVL = 45.5, TL = 37.4, TW = 5.2, LAL = 6.1, CL = 7.8, AGL = 18.6, BH = 4.2, BW = 8.9, HL = 12.0, HW = 8.7, HD = 5.3, ED = 2.9, EE = 3.8, ES = 5.3, EN = 3.8, IN = 1.9, IO = 3.0, EL = 1.4. Meristic data: INS = 1, SL L&R = 12&9, IL L&R = 9&10, SL M L&R = 7&6, IL M L&R = 5&6, PVT L&R = 30&31, DTR = 18, MVSR = 32, VS1 = 50, VS2 = 150, DLAMF1 L&R = 7&6, BLAMF1 L&R = 3&2, DLAMF4 L&R = 7&8, BLAMF4 L&R = 6&5, DLAMT1 L&R = 8&8, BLAMT1 L&R = 2&2, DLAMT4 L&R = 8&8, BLAMT4 L&R = 7&7, DLAMT5 L&R = 10&9, BLAMT5 L&R = 6&6, TLAMF1 L&R = 10&8, TLAMF4 L&R = 13&13, TLAMT1 L&R = 10&10, TLAMT4 L&R = 15&15, TLAMT5 L&R = 16&15, and PCT L&R = 2&2. Colouration and pattern darker and more defined, five pairs of dorsal spots with two much smaller pairs following the 2^nd^ and 3^rd^ spot; limbs with dark reticulations, ~ 9 dark bands on tail, ventral surfaces with many more dark spots and streaks (Fig. 9A, B).

###### Etymology.

The specific epithet is a toponym for the Terai, a unique lowland habitat in southern Nepal and north India, bordered by the Siwalik Range in the north and the Indo-Gangetic Plains to the south. The suggested common name is Terai clouded *Geckoella*/ ground gecko.

###### Distribution and natural history.

Cyrtodactylus (Geckoella) teraiensis sp. nov. is the first ground-dwelling bent-toed gecko described from Nepal. The species is known only from its type locality in the Belbari Chisang Collaborative Forest, Bhaunne, Morang District, Koshi Province (Fig. 1). The site is within the Dharan Forests and is denoted as both an Important Bird Area (IBA) and a Key Biodiversity Area (KBA) (BCN, DNPWC, DOFSC 2024). This location is south of the Siwalik Range, where hilly country transitions to the flat, low-elevation Gangetic floodplain, and falls within the Terai-Duar Savanna and Grasslands ecoregion (Olson et al. 2001; Olson and Dinerstein 2002), characterised by Sal dominant forest. The habitat consists of dense, closed-canopy forest with abundant understory bushes (Fig. 8B). Two individuals were recorded on 27 April 2025 between 2130–2200 hrs. The holotype was found crossing the gravel-surfaced Bhaunne-Letang road, the edges of which were covered in Sal leaf litter and scattered grass (5–10 cm height). The paratype was observed on a forest trail amidst leaf litter and grass. The two observation points were ~560 m apart. We were unable to record any more specimens during subsequent nighttime surveys in May, July, and August. Syntopic lizards observed in the area included the geckos *Hemidactylus* sp., *Gekko gecko* (Linnaeus, 1758), and *Calotes cf. versicolor*. The type locality is subject to low-level anthropogenic pressure, primarily from collection of non-timber forest produce such as fodder, grass, and edible ferns (pers. obs.). However, one of the major potential threats to the habitat is forest fire as 56 forest fires have been detected in the last 10 years within 3 km radius of the type locality (NASA 2025).

## Discussion

*Cyrtodactylus teraiensis* sp. nov. is the 18^th^ species of *Geckoella*, the second species of the C. (Geckoella) nebulosus complex, and the first *Geckoella* from Nepal and north of the Tropic of Cancer. This extends the range of *Geckoella* by ~500 km northward and, significantly, across two potential dispersal barriers — the Indo-Gangetic Plains (biogeographic barrier) and the Tropic of Cancer (climatic barrier). The *C. nebulosus* complex in India is predominantly distributed in dry and moist deciduous to evergreen forest in hilly areas from ~ 100– > 1200 m a.s.l. and is separated from the Nepal population in the Terai-Duar by the Indo-Gangetic Plains. The Indo-Gangetic Plains are low elevation alluvial deposits with little relief that form a divide between peninsular Indian and Southeast Asian/ Himalaya biota (Mani 1974; Morley 2018). Originally dominated by grasslands with small fragments of deciduous forest, the vast majority of this densely populated region has been converted for agricultural use or otherwise degraded (Rattan et al. 2021). The Indo-Gangetic Plains had already formed and were expanding by the early Miocene and likely functioned as a biogeographic barrier (Mani 1974; Singh 1996). The timing of divergence between *Cyrtodactylus teraiensis* sp. nov. and Indian members of the *C. nebulosus* complex in the middle-late Miocene overlaps with the Mid-Miocene Climatic Optimum, a time of global warmth during which tropical forests expanded (Morley 2011, 2018) creating dispersal corridors for forest-adapted taxa between the Indian subcontinent and Southeast Asia (Klaus et al. 2016). It is possible that other forest-restricted or cool-adapted groups that are currently considered endemic to peninsular India may extend into the Terai-Duar or Siwaliks with a similar mid-Miocene time of divergence from peninsular Indian congeners – potentially the Eastern Ghats clade of *Hemiphyllodactylus* and some clades of Indian *Hemidactylus* that occur in the northern Eastern Ghats (Agarwal et al. 2019a, 2019b).

The region of eastern Nepal is also the first area in South Asia where *Cyrtodactylus* species from two divergent species groups (Grismer et al. 2021) are likely to overlap in distribution – *C. martinstolli* (Darevsky, Helfenberger, Orlov & Shah, 1998) of the Indo-Burma clade has been collected within ~10 km of the new species (Bhattarai et al. unpub. data) — although they are yet to be recorded in sympatry. The recent discoveries of new *Cyrtodactylus* of the Indo-Burma clade from across Nepal are not surprising and many more new species are likely to be discovered with additional survey effort (e.g. Bhattarai et al. 2025a, 2025b; Subba et al. 2025). Though the Nepalese *Geckoella* diverged from Indian populations in the mid-Miocene, it remains to be seen if this group has speciated or dispersed east/ west in the Terai-Duar or Siwaliks. It is clear that the *C. nebulosus* group is a large species complex in peninsular India and a taxonomic revision of the group is in progress (Agarwal, Khandekar, Thackeray unpubl. data). Most *Geckoella* species are members of closely related species complexes – and only *C. jeyporensis* and *C. teraiensis* sp. nov. are relict taxa, the former distributed in high elevations of the Eastern Ghats (Agarwal et al. 2012; Agarwal and Karanth 2015).

Endemic squamate radiations within peninsular India can be broadly divided into Western Ghats endemics (e.g. gekkonids *Dravidogecko* and numerous clades of South Asian *Cnemaspis*, snakes such as *Teretrurus* Beddome, 1886, *Melanophidium* Günther, 1864, *Xylophis* Beddome, 1878), tropical peninsular India endemics (e.g. gekkonids *Calodactylodes*, numerous *Cnemaspis* clades, Cyrtodactylus (Geckoella), *prashadi* group of Indian *Hemidactylus*, *Hemiphyllodactylus*; the scincid *Dravidoseps*; and snakes of the genus *Uropeltis* Cuvier, 1829), and finally endemics to the subcontinent (e.g. *brookii* group of Indian *Hemidactylus* geckos) (Bansal and Karanth 2010; Bauer et al. 2010; Agarwal and Karanth 2015; Agarwal et al. 2019a, 2019b, 2020, 2024; Chaitanya et al. 2019; Pal et al. 2021, Sampaio et al. 2023, Cyriac et al. 2024). This last category also includes the agamids *Sitana* Cuvier and *Sarada* Deepak, Giri and Karanth and the small-bodied clade of the lacertid *Ophisops* Ménétries, 1832, though these are both diurnal taxa restricted to open habitats (Agarwal and Ramakrishnan 2017; Deepak and Karanth 2018). In addition, the skinks *Eutropis* Fitzinger, 1843 and *Riopa* (Gray, 1839) have endemic radiations in the subcontinent and Southeast Asia (Datta-Roy et al. 2012, 2014). *Cyrtodactylus teraiensis* sp. nov. is the first example of a Terai-Duar endemic species from a group otherwise restricted to peninsular India. This discovery not only extends the known distribution of Cyrtodactylus (Geckoella) northward across a significant biogeographic divide but also reinforces the need for targeted research across under-surveyed landscapes in Nepal and India.

## Supplementary Material

XML Treatment for Gymnodactylus nebulosus

XML Treatment for Cyrtodactylus (Geckoella) teraiensis

## Additional information

### Use of AI

No use of AI was reported.

### Author ORCIDs

Bivek Gautam https://orcid.org/0000-0001-5253-7801

Santosh Bhattarai https://orcid.org/0000-0001-7615-1954

Bishal Prasad Neupane https://orcid.org/0009-0000-3450-5195

Chiranjibi Prasad Pokheral https://orcid.org/0000-0001-7252-1946

Tejas Thackeray https://orcid.org/0000-0002-9981-8763

Akshay Khandekar https://orcid.org/0000-0002-7956-089X

Vivek Phillip Cyriac https://orcid.org/0000-0002-0465-0452

Ishan Agarwal https://orcid.org/0000-0001-9734-5379

### Data availability

All of the data that support the findings of this study are available in the main text.
